# Recognizing mid-career productivity: the 2008 *Retrovirology *Prize, call for nomination

**DOI:** 10.1186/1742-4690-5-80

**Published:** 2008-09-11

**Authors:** Kuan-Teh Jeang

**Affiliations:** 1 The National Institutes of Health, Bethesda, MD, USA

## Abstract

A recent analysis suggested a narrow age range for productivity of innovative work by researchers. The *Retrovirology *Prize seeks to recognize the research of a mid-career retrovirologist between the ages of 45 and 60. The 2007 *Retrovirology *Prize was awarded to Dr. Karen Beemon. Nominations are being solicited for the 2008 prize.

## Background

In the August 18th, 2008, *Wall Street Journal*, George Anders wrote a column on the measures being taken by high technology companies in the United States to extend their researchers' age span of productivity. Anders cited an analysis performed by Benjamin Jones, a professor at Northwestern University's Kellogg School of Management, on innovative breakthroughs. Jones was reported to have examined the biographical data of more than 700 Nobel laureates and renowned researchers of the past century. One the conclusions reached by Jones was that "innovators are productive over a narrowing span (of approximately 25 years) of their life cycle" with researchers being most productive between the ages of just before 30 to 55, peaking at age 40. Reflecting the notion that innovative research often comes in early- to mid-career, *Retrovirology *seeks to recognize the work of a deserving retovirologist between the ages of 45 and 60 with the *M. Jeang Retrovirology Prize *[1].

**Figure 1:**
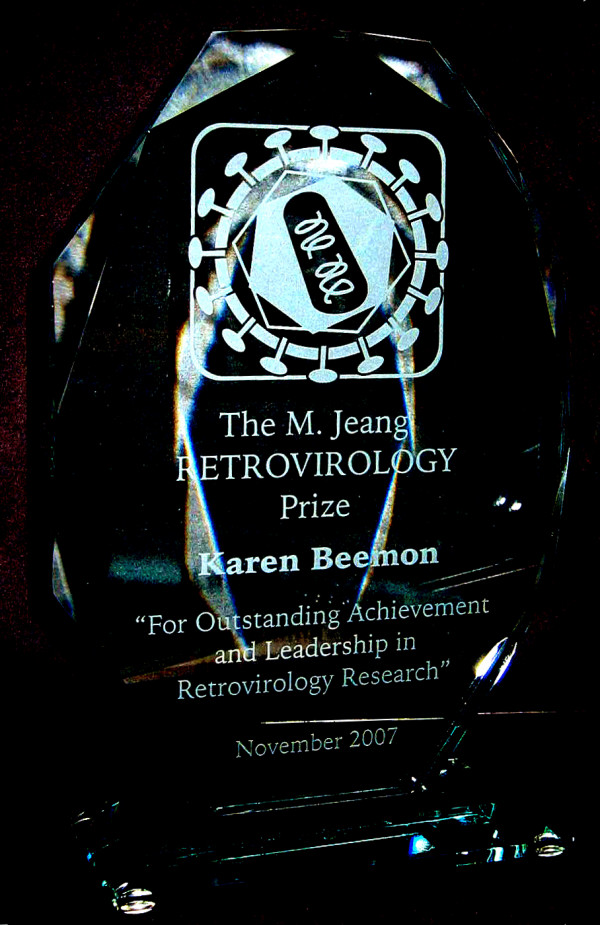
A photograph of the crystal trophy presented to Dr. Karen Beemon, winner of the 2007 M. Jeang *Retrovirology *Prize.

Since its inception, the Retrovirology prize has been awarded three times, in 2005 to Stephen Goff [2], in 2006 to Joseph Sodroski [3], and in 2007 to Karen Beemon [4]. The Prize consists of an attractive crystal trophy (Figure 1), a $3,000 cash award, and a profile article of the awardee published in *Retrovirology*. The *Retrovirology *Prize is supported, in part, through a donation from the Ming K. Jeang Foundation, an educational foundation based in Houston, Texas, USA.

## Call for nominations and the selection process

As stated previously [1], the Prize alternates yearly between recognizing a non-HIV retrovirologist (2007 and odd years) and an HIV retrovirologist (2008 and even years). There can be some discretion on this guideline, exercised from time-to-time by the selection committee. Any individual can initiate a nomination of others or self-nominate. A nomination includes a statement (1000 words or less) of the nominee's significant contributions to retrovirus research, a curriculum vitae of the nominee, and a statement by the nominator that the nominee has agreed to be nominated. The selection committee consists of the Editors of *Retrovirology *(currently, M. Benkirane, B. Berkhout, M. Fujii, K.T. Jeang, M. Lairmore, A. Lever, and M. Wainberg). All nominations submitted to the selection committee must be communicated through an Editorial Board member of *Retrovirology*. Hence, an individuals who is not an Editorial board member but who wishes to make a nomination should seek out a *Retrovirology *Editorial board member to communicate his/her information to the selection committee. A list of current Editorial Board members can be found at the *Retrovirology *website . Within the stipulated age limits, all *Retrovirology *Editors and Editorial Board members are eligible to be nominated with the exception of the Editor-in-Chief who will administer the final selection.

For 2008, nominations will be accepted beginning on September 15^th ^and will close on October 30^th^. All members of the retrovirology community are encouraged to participate in this process for recognizing a deserving colleague.
